# Prostate tissue microbiota dysbiosis associated with prostate cancer: results from the MICROPROSTK multicenter study

**DOI:** 10.1016/j.nmni.2026.101862

**Published:** 2026-10-08

**Authors:** R. Lotte, A. Gaudart, C. Cruzel, S. Droupy, J.P. Lavigne, F. Bruyère, P. Lanotte, E. Lechevallier, G. Dubourg, P.E. Fournier, R. Haider, L. Lotte, K. Youssef, A. Emery, R. Madany, M. Payen, L. Pfauwadel, B. Tibi, Y. Ahallal, S. Zouari, I. Bentellis, E. Fontas, M. Durand, R. Ruimy

**Affiliations:** a Laboratoire de Bactériologie du CHU de Nice, Hôpital Archet 2, Nice, France; b Inserm U1065, Centre Méditerranéen de Médecine Moléculaire, Équipe 6, Nice, France; c Université Cote d’Azur, Faculté de Médecine de Nice, Nice, France; d Délégation à la Recherche Clinique et à l’Innovation du CHU de Nice, Hôpital de Cimiez, Nice, France; e Service d’Urologie du CHU de Nîmes, Hôpital Carémeau, Nîmes, France; f Laboratoire de Microbiologie du CHU de Nîmes, Hôpital Carémeau, Nîmes, France; g Service d’Urologie du CHU de Tours, Hôpital Bretonneau, Tours, France; h Laboratoire de Bactériologie du CHU de Tours, Hôpital Bretonneau, Tours, France; i Service d’Urologie de l’APHM, Hôpital la Conception, Marseille, France; j Institut Hospitalo-Universitaire Méditerranée Infection, Marseille, France; k Service d’Urologie du CH de Cannes, Hôpital Simone Veil, Cannes, France; l Laboratoire de Biologie du CH de Cannes, Hôpital Simone Veil, Cannes, France; m Service d’Urologie du CH de Menton, Hôpital La Palmosa, Menton, France; n Service d’Urologie du CHU de Nice, Hôpital Pasteur 2, Nice, France

**Keywords:** Prostate microbiota, Prostate cancer, Microbial dysbiosis, 16S rDNA sequencing, Pathobionts

## Abstract

**Background:**

The prostate is a poorly characterized microbial niche. Bacterial communities may contribute to prostate carcinogenesis through chronic inflammation. We aimed to describe the prostate microbiota at species level and identify bacteria associated with prostate cancer (PCa).

**Methods:**

MICROPROSTK is a prospective, multicenter study including 235 men undergoing initial prostate biopsy (122 PCa, 113 non-PCa). Urine, prostate tissue, and rectal swabs were collected. Full-length 16S rDNA sequencing was used for species-level identification. Comparative microbiota analyses were performed in 141 patients with complete datasets. Species richness, α-diversity, β-diversity, and differential abundance of taxa were assessed.

**Results:**

A total of 959 bacterial species were identified in prostate tissue, supporting the existence of a diverse microbial ecosystem. PCa prostate tissue samples showed higher richness and α-diversity than tissue from non-PCa patients (p = 0.046 and p = 0.019) while urine and rectal microbiota showed no significant differences in these metrics. β-diversity analysis confirmed distinct microbial compositions across anatomical sites. Several species were enriched in PCa, including *Cutibacterium acnes*, *Eubacterium rectale*, *Bacteroides dorei*, and *Ruminococcus torques*, whereas *Lactobacillus reuteri* was enriched in non-PCa patients.

**Conclusions:**

Our multicenter study shows that the prostate harbors a specific microbiota with cancer-associated dysbiosis. The identification of specific bacterial species supports a potential role for pathobionts in PCa and highlights candidate microbial biomarkers. These findings expand current knowledge of human-associated microbial niches and warrant further investigation into their clinical and pathogenic significance.

## Highlights

• The prostate harbors a diverse and previously undercharacterized microbial ecosystem with species-level resolution.
• Prostate cancer (PCa) patients exhibit higher microbial richness and α-diversity in prostate tissue than non PCa patients.
• Distinct microbial communities are observed across prostate, urinary, and rectal niches, independent of cancer status.
• *Cutibacterium acnes* is enriched in PCa patients' biopsies, *Lactobacillus reuteri* in non-PCa patients' biopsies.
• Findings highlight potential prostate-associated pathobionts and protective commensalssignatures.

## 1 Introduction

Prostate cancer (PCa) is the second most frequently diagnosed cancer and the fifth leading cause of cancer-related death among men worldwide. In Europe, 473 011 new cases and 115 182 deaths were estimated in 2022, while in France, 50 400 new cases were reported in 2018. Despite major advances in early detection and treatment that have improved survival, these interventions often compromise sexual function and overall quality of life [1,2].

The mechanisms driving PCa initiation and progression remain incompletely understood and no specific PCa prevention strategy can be recommended at this time [1]. Both genetic and acquired factors—including diet, lifestyle, and chronic inflammation—have been implicated in PCa pathogenesis [3,4]. Microbial infections are increasingly recognized as potential contributors to carcinogenesis, as evidenced by *Helicobacter pylori* in gastric cancer and human papillomavirus in cervical cancer [5,6], suggesting that microbial dysbiosis may also play a role in prostate carcinogenesis.

Recent advances in sequencing technologies have challenged the assumption that urine is sterile, revealing a urinary microbiota capable of interacting with the immune system and modulating local inflammation, potentially contributing to prostate pathology [[7], [8], [9], [10], [11], [12]]. Studies of prostate tissue microbiota are emerging, but most have been limited by small cohorts and the use of short-read 16S rDNA sequencing, which restricts taxonomic resolution to the genus level [[13], [14], [15], [16], [17], [18]].

To address these limitations, we conducted a multicenter, standardized, prospective study to characterize the prostate microbiota using full-length 16S rDNA sequencing (PacBio), allowing species-level identification of amplicon sequence variants (ASVs). Our primary aim was to compare the biodiversity of prostate microbiota on biopsy samples between PCa patients and non-PCa patients. Secondary objectives included the comparison of urinary and rectal microbiota. These analyses may help identify specific bacterial signatures associated with PCa and inform the development of microbial biomarkers for early detection and risk stratification.

## 2 Materials and methods

### 2.1 Study design and cohort

MICROPROSTK is a national, prospective, multicenter study investigating the prostatic, urinary, and rectal microbiota in men with or without biopsy-confirmed PCa, including early-stage patients. The trial (NCT03947515) was approved by the institutional ethics committee (IDRCB: 2019-A00741-56) and conducted in accordance with the Declaration of Helsinki. Written informed consent was obtained from all participants. Patients were recruited from six French hospitals between February 2021 and June 2023.

### 2.2 Patient enrollment and sampling

Men with suspicion of PCa based on abnormal digital rectal examination (DRE), prostate-specific antigen (PSA) elevation, or magnetic resonance imaging (MRI) findings were screened. Exclusion criteria included recent antibiotic use (3 months), recent uro-genital surgery, or contraindications to biopsy. Urine samples were collected after DRE, followed by prostate biopsies and rectal swabs. All participants received a single oral dose of prophylactic fluroquinolones (ciprofloxacin 500 mg) 2 h before biopsy. Patients were classified as PCa or non-PCa based on histology.

### 2.3 Clinical data

Demographic, clinical, imaging, and histopathological data were recorded, including age, Charlson's comorbidity index, PSA, MRI findings, biopsy results, and cancer grading (ISUP and Gleason).

### 2.4 Microbiota analysis

DNA was extracted from urine, prostate biopsies and rectal swab using standardized protocols. Microbiome profiling was performed using full-length 16S rDNA SMRT sequencing (PacBio Sequel II HiFi). Reads were processed into Amplicon Sequence Variants (ASVs) for taxonomic assignment, and α- and β-diversity metrics were calculated. Differences between PCa and non-PCa patients were analyzed statistically. Given the low biomass nature of prostate biopsy samples, particular attention was paid to minimizing potential contamination. Standardized sampling procedures were applied across all centers, and DNA extraction and sequencing were performed using uniform protocols. A blank control was included in each DNA extraction batch to monitor potential contamination during DNA extraction and sample processing. These blank controls were then amplified and sequenced. Then stringent quality filtering and downstream bioinformatic processing were applied to reduce the impact of potential contaminants. **Note:** Detailed protocols for sample handling, DNA extraction, library preparation, sequencing, and bioinformatics are provided in the **Supplemental Materials**.

### 2.5 Statistical analysis

Baseline characteristics were described and compared between PCa and non-PCa patients using SAS Enterprise Guide software (version 7.1; SAS Institute Inc., Cary, NC, USA). The Chi-square test (or Fisher's exact test for small sample size) and Student t-test (or the Wilcoxon rank sum test for non-normally distributed data) were respectively used to compare qualitative and quantitative variables between the two groups.

## 3 Results

### 3.1 Study population

Between February 2021 and June 2023, 296 patients were enrolled across six tertiary care centers. Of these, 61 (20.6%) did not complete the study: 17 had received antibiotics within the three months preceding biopsy, 16 patients discontinued participation after enrollment, 12 deviated from the study protocol, 9 were lost to follow-up, and 5 were excluded at the investigator's discretion, 2 withdrew consent. Ultimately, 235 patients were included in the final analysis. A total of 705 samples were obtained from these patients (235 urine samples, 235 prostate biopsies, and 235 rectal swabs). Adequate DNA yield for microbiota analysis from all three sample types was available in 141 patients, who were included in the comparative analysis between PCa and non-PCa patients.

### 3.2 Clinical and paraclinical characteristics

Clinical data were available for the 235 patients who completed the study (Table 1a). Of these, 122 (51.9%) were diagnosed with PCa, while 113 (48.1%) had no evidence of malignancy on biopsies and were classified as non-PCa patients.

**Table 1a:** Main characteristics of Cancer and Non-Cancer groups among the 235 patients who completed the MICROPROSTK study.

		PCa group	Non-PCa group	Statistical analysis
Number of patients		122	113	
Age		70.0±6.8	67.7±7.2	p = 0.012*
Charlson's comorbidity index		3.2±1.4	2.8±1.5	p = 0.007**
Past family history of PCa		18/122 (14.7%)	11/113 (14.8%)	p = 0.242+
Abnormal digital rectal examination		40/119 (33.6%)	36/108 (33.3%)	p = 0.964+
Total PSA (ng/mL)		12.1± 13.7	9.4±8.2	p = 0.178**
Mean prostate volume (cm3)		54.7± 27.9	71.3±43.2	p = 0.0022**
Largest diameter of MRI index lesion (mm)		14.2±7.4	15.7±7.6	p = 0.125**

PIRADS score	**I**	0/91 (0%)	1/64 (1.6%)	**p = 0.002++**
	**II**	2/91 (2.2%)	3/64 (4.7%)	
	**III**	10/91 (11.0%)	18/64 (28.1%)	
	**IV**	43/91 (47.3%)	31/64 (48.4%)	
	**V**	36/91 (39.5%)	11/64 (17.2%)	
Anatomic localization of prostatic MRI lesion	**Base**	25/95 (26.3%)	19/64 (29.7%)	p = 0.866+
	**Median**	34/95 (35.8%)	23/64 (35.9%)	
	**Apex**	36/95 (37.9%)	22/64 (34.4%).	
	**Right Lobe**	44/94 (46.8%)	31/65 (47.7%)	p = 0.913+
	**Left Lobe**	50/94 (53.2%)	34/65 (52.3%)	
	**Median**	51/90 (56.7%)	37/63 (58.7%)	p = 0.799+
	**Lateral**	39/90 (43.3%)	26/63 (41.3%)	
MRI detected EPE		10/96 (10.4%)	3/65 (4.6%)	p = 0.185+
PIN Lesion on biopsies			4/113 (3.5%)	
Inflammation on biopsies of non-PCa patients			36/113 (31.8%)	
No specific histopathological lesion			93/113 (82.3%)	
ECE on biopsies in PCa patients		4/120 (3.3%)		
PNI on biopsies in PCa patients		11/121 (9.1%)		

ISUP Grade for PCa group	**1**	49/119 (41.2%)		
	**2**	35/119 (29.4%)		
	**3**	10/119 (8.4%)		
	**4**	17/119 (14.3%)		
	**5**	8/119 (6.7%)		

Patients with PCa were older than non-PCa (mean age: 70.0 ± 6.8 vs 67.8 ± 7.2 years, *p* = 0.012) and had a higher Charlson comorbidity index (3.2 ± 1.4 vs 2.8 ± 1.5, *p* = 0.007). A family history of PCa was reported in 29 patients (12.3%) without significant difference between groups. Abnormal DRE was observed in 76 patients (33.5%), with comparable rates between groups. Mean total PSA level was 10.75 ± 11.4 ng/ml and did not differ significantly between PCa and non-PCa patients.

Prostate MRI showed a larger prostate volume in non-PCa patients compared with PCa patients (71.3 vs 54.7 cm^3^, *p* = 0.002). The mean size of the largest suspicious lesion was 14.8 mm, with no intergroup difference (*p* = 0.125). PI-RADS scores differed significantly between groups (*p* = 0.0024). In patients undergoing MRI, the index lesion was most frequently located at the apex (36.5%), in the left lobe (52.8%), and in the mid-gland (57.5%), with no difference in distribution between groups. Extra-prostatic extension was detected on MRI in 8.1% of cases, again without intergroup difference.

Histological examination in PCa patients most commonly revealed ISUP grade 1 tumors (41.2%), while extracapsular extension and perineural invasion were present in 3.3% and 9.1% of cases, respectively. In non-PCa patients, prostatic intraepithelial neoplasia and prostatitis were identified in 3.5% and 31.8% of patients, respectively.

After excluding samples with insufficient sequencing depth and performing quality filtering and denoising, we characterized the bacterial community composition across sample types from 141 patients of whom 72 (51.1%) had PCa and 69 (48.9%) were non-PCa. Demographic, clinical and paraclinical characteristics in this subgroup were comparable to those of the overall study population, including the proportion of non-PCa patients presenting with histological prostatitis (22/69, 31.9%). Detailed data for this subgroup are provided in Table 1b.

**Table 1b:** Main characteristics of Cancer and non-Cancer groups among the 141 patients eligible for comparative microbiota analysis.

		PCa group	Non PCa group	Statistical analysis
Number of patients		72	69	
Age		70.0±7.3	67.0±7.6	p = 0.016*
Charlson's comorbidity index		3.2±1.3	2.8±1.6	p = 0.016**
Past family history of PCa		12/72 (16.7%)	8/69 (11.6%)	p = 0.388+
Abnormal digital rectal examination		27/69 (39.1%)	21/66 (31.8%)	p = 0.375+
Total PSA (ng/mL)		12.8± 15.9	9.2±9.2	p = 0.092**
Mean prostate volume (cm3)		55.3± 27.9	70.1±47.0	p = 0.002**
Largest diameter of MRI index lesion (mm)		12.7±5.5	16.2±9.1	p = 0.067**

PIRADS score	**I**	0/51 (0%)	0/38 (0%)	**p = 0.009++**
	**II**	1/51 (2.0%)	1/38 (2.6%)	
	**III**	4/51 (7.8%)	12/38 (31.6%)	
	**IV**	25/51 (49.0%)	18/38 (47.4%)	
	**V**	21/51(41.2%)	7/38 (18.4%)	
Anatomic localization of prostatic MRI lesion	**Base**	15/52 (28.8%)	10/39 (25.7%)	p = 0.715+
	**Median**	17/52 (32.7%)	16/39 (41.0%)	
	**Apex**	20/52 (38.5%)	13/39 (33.3%).	
	**Right Lobe**	24/52 (46.2%)	19/39 (48.7%)	p = 0.804+
	**Left Lobe**	28/52 (53.8%)	20/39 (51.3%)	
	**Median**	31/49 (63.3%)	22/38 (57.9%)	p = 0.611+
	**Lateral**	18/49 (36.7%)	16/38 (42.1%)	
MRI detected EPE		5/53 (9.4%)	2/37 (5.1%)	p = 0.695+
PIN Lesion on biopsies			2/69 (2.9%)	
Inflammation on biopsies of Non-PCa patients			22/69 (31.9%)	
No specific histopathological lesion			56/69 (81.2%)	
ECE on biopsies in PCa patients		3/72 (4.2%)		
PNI on biopsies in PCa patients		7/72 (9.7%)		

ISUP Grade PCa group	**1**	29/71 (40.8%)		
	**2**	20/71 (28.2%)		
	**3**	5/71 (7.0%)		
	**4**	11/71 (15.5%)		
	**5**	6/71 (8.5%)		

PCa: prostate cancer, Non-PCa: absence of prostate cancer on biopsy, PSA: Prostate Specific Antigen; MRI: Magnetic Resonance Imaging; PIRADS: Prostate Imaging Reporting and Data System; ISUP: International Society of Urological Pathology; EPE: Extraprostatic Extension on MRI; PIN: Prostatic Intraepithelial Neoplasia; ECE: Extracapsular Extension on biopsy; PNI: Perineural Invasion.

p values were calculated by using different statistical test: Student t-test (*), Wilcoxon-rank sum test (**), Chi-square test (+) and the Fisher's exact test(++).

### 3.3 Bacterial community structure and composition

#### 3.3.1 Sequencing characteristics, α- and β-diversity in PCa and non-PCa groups

In total, 11 922, 3 741, and 15 424 ASVs were identified in prostate biopsy, urine, and rectal samples, respectively. The α-diversity metrics represented by the Shannon index, for prostate, urine, and rectal samples are shown in Fig. 1. In prostate biopsies, both species richness and Shannon diversity were significantly higher in PCa patients compared with non-PCa (*p* = 0.046 and *p* = 0.019, respectively). Although no significant differences were observed in urine or rectal samples (*p* > 0.05), both indices tended to be higher in PCa patients. Across sample types, urine consistently showed the lowest richness and diversity (Fig. 1). No significant differences in terms of beta diversity were detected between PCa and non-PCa patients in any sample type (Fig. 2). However, differences were observed between prostate, urine, and rectal microbiota, confirming that the prostate harbors a distinct microbial community (data not shown).

**Fig. 1:**
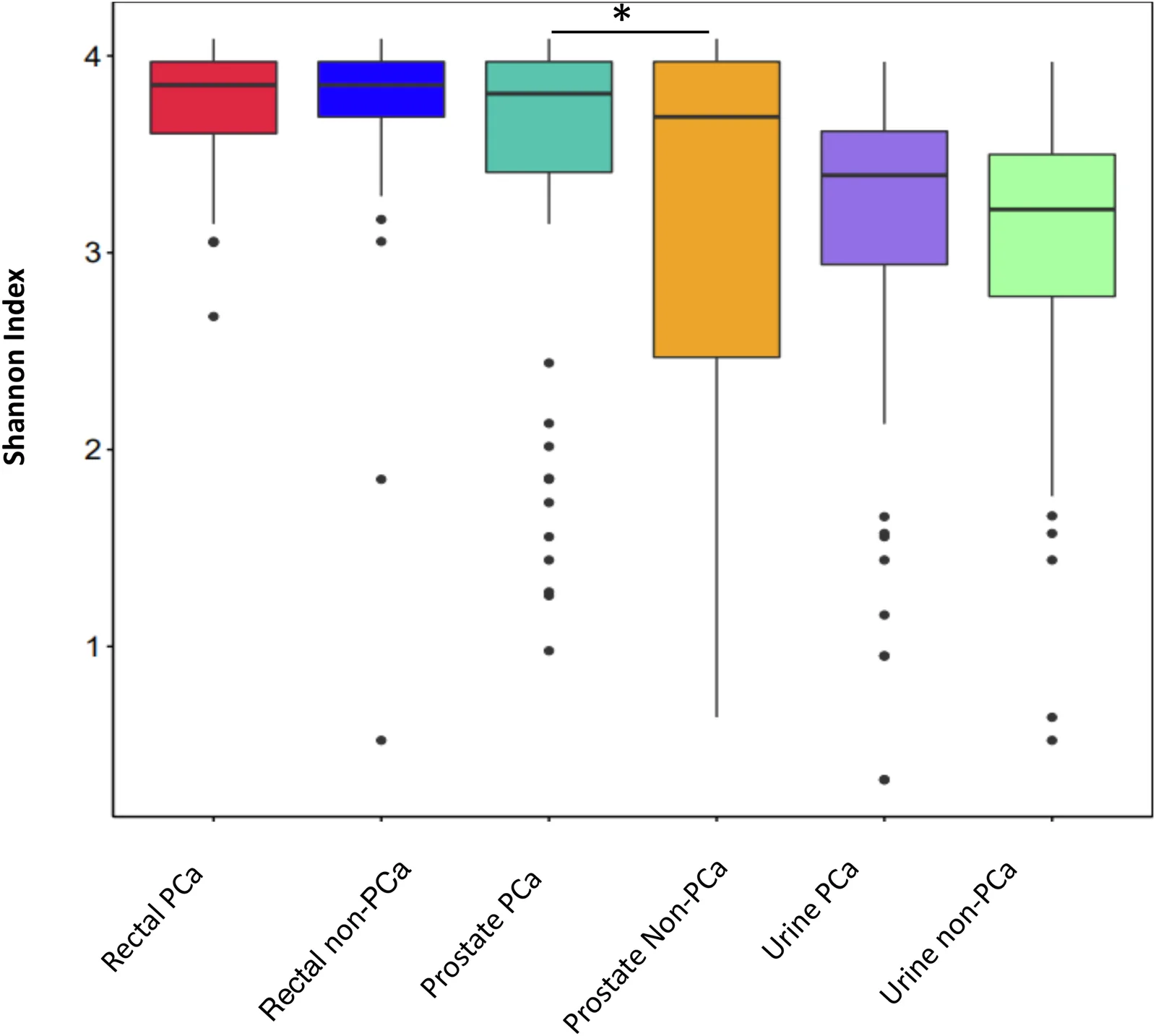
**Alpha diversity metrics evaluated by Shannon index comparison between PCa and non-PCa patients in each type of biological samples** Box plots of alpha diversity (Shannon Index) in rectal, prostate biopsies and urine microbiota for PCa and non-PCa patients. A Kruskall Wallis test was used to compared Shannon index between patients' group.

**Fig. 2:**
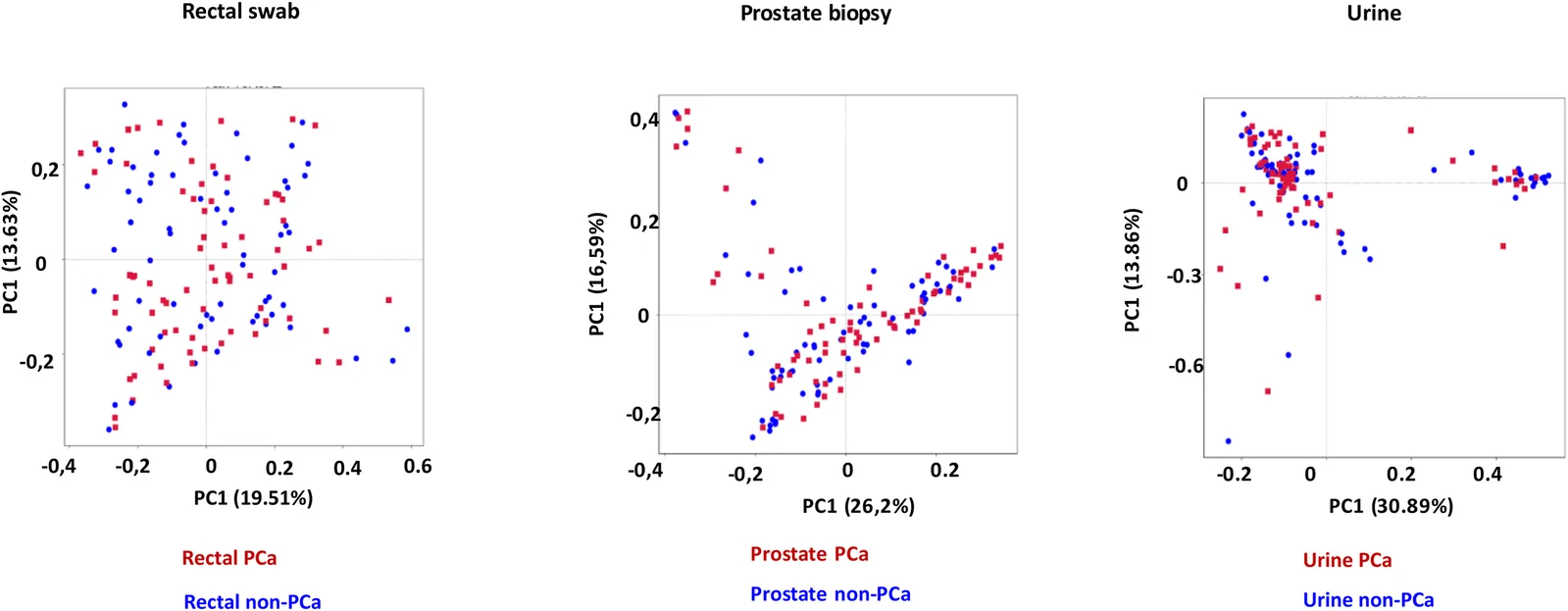
**Beta diversity metrics evaluated using Unifrac distances and visualized by principal coordinate analysis (PCoA) for PCa and non-PCa patients in each type of biological samples** β-diversity is represented using principal coordinate analysis (PCoA) plot based on Unifrac distance matrices with respect to the bacterial composition among PCa and non-PCa patients in each type of sample.

#### 3.3.2 Taxonomic composition of prostate biopsy samples

We next characterized the taxonomic composition of prostate biopsy samples, providing for the first time a species-level description of the prostatic microbiota. Overall, 22 phyla, 31 classes, 83 orders, 158 families, 330 genera, and 959 species were identified. The five most abundant phyla in both PCa and non-PCa patients were *Firmicutes, Bacteroidota, Cyanobacteriota, Proteobacteria, and Actinobacteria*. At the class level, *Clostridia, Bacteroidia, Cyanobacteria, Bacilli, and Gammaproteobacteria* predominated.

The most abundant bacterial genera across all prostate samples were *Prevotella, Bacteroides, Faecalibacterium, Ruminococcus, Streptococcus, Agathobacter, Lactobacillus, Campylobacter, and Fenollaria*. At the bacterial species level, the most abundant taxa included *Ruminococcus* spp., *Faecalibacterium prausnitzii, Eubacterium rectale, Campylobacter hominis, Streptococcus* spp., *Akkermansia muciniphila, Eubacterium coprostanoligenes, Prevotella copri,* and *Blautia* spp.

#### 3.3.3 Differential abundance between PCa and non-PCa patients

At the phylum level, no major differences were observed except for *Synergistota*, which was enriched in PCa patients (*p* = 0.026). At the class level, *Clostridia* and *Synergistia* were significantly more abundant in PCa patients. At the genus level, *Bacteroides*, *Agathobacter*, *Ruminococcus****,*** *Cutibacterium*, and *Lachnoclostridium* were enriched in PCa, whereas *Chryseobacterium* was more abundant in non-PCa patients.

Species-level analysis using full-length 16S rDNA sequencing revealed that *Eubacterium rectale, Cutibacterium acnes, Bacteroides dorei, Ruminococcus torques, Bacteroides massiliensis, Ruminococcus lactaris, Eubacterium halii, Ruminococcus sp., Bilophila wadsworthia, Clostridium disporicum,* and *Clostridium spiroforme* were significantly enriched in PCa patients. Conversely, *Lactobacillus reuteri, Chryseobacterium echinoideorum, and Candidatus Arthromitus* were significantly more abundant in the non-PCa (Fig. 3). Of note, five of the previously mentioned species exhibited a mean relative abundance greater than 1% in at least one of the two groups (PCa and non-PCa patients). These were, in order of abundance, *E*. *rectale*, *C*. *acnes*, *B*. *dorei*, *L. reuteri*, and *R*. *torques*. These findings highlight candidate species that may be of interest for future validation studies.

**Fig. 3:**
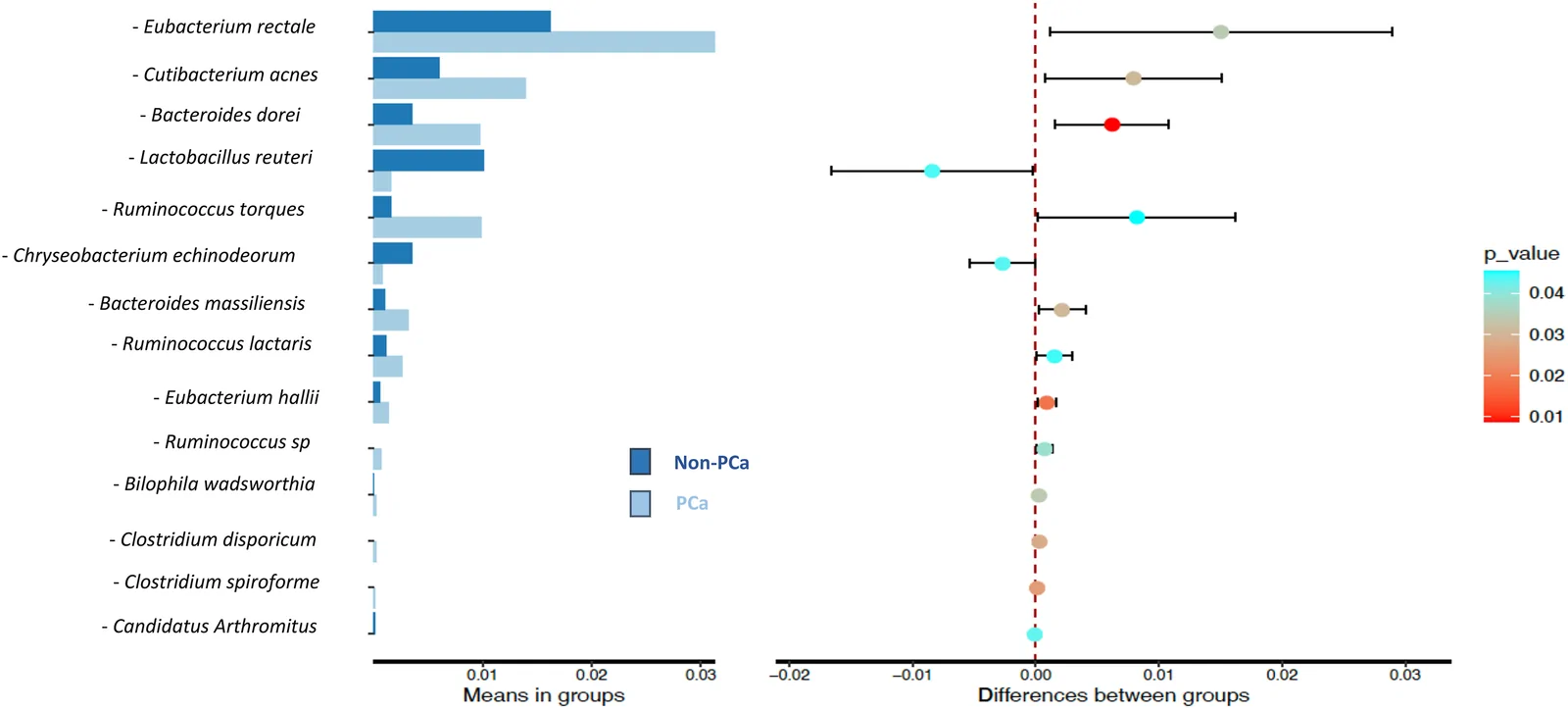
**Differential abundance of species in prostate biopsy samples between PCa and non-PCa patients** The left figure shows the species abundance differences between groups, and each bar in the figure represents the average abundance of the species in different groups; the right figure shows the confidence of inter-group differences. The leftmost point of each circle in the figure represents the lower limit of 95% confidence interval, and the rightmost point of the circle represents the upper limit of 95% confidence interval. The center of the circle represents the differences of the mean value, and the color of the circle represents the P value of differences significance test between groups of the corresponding species.

Data on urinary and fecal microbiota are provided in the **Supplemental materials.**

## 4 Discussion

The MICROPROSTK study provides, to our knowledge, the first large multicenter prospective characterization of the prostate microbiota at species-level resolution in men with and without prostate cancer. Using full-length 16S rDNA sequencing, we identified distinct microbial signatures associated with PCa, with several bacterial species significantly enriched in cancer tissues.

Our cohort, recruited across six tertiary care centers, reflects real-world clinical practice. As expected, patients with PCa were older and had a higher Charlson comorbidity index, consistent with known epidemiological data. Although PSA levels, abnormal digital rectal examination findings, and family history were more frequent in the PCa patients, these differences were not statistically significant, highlighting the limitations of current diagnostic tools and the need for complementary biomarkers. The predominance of low-grade tumors (ISUP 1) suggests that our findings mainly reflect early-stage disease, which is particularly relevant for biomarker discovery but may limit extrapolation to more aggressive PCa.

A key finding of our study is the significantly higher richness and α-diversity observed in prostate tissue from PCa patients. This contrasts with several previous reports and may be explained by differences in cohort size, sequencing depth, and taxonomic resolution [[17], [18], [19]]. Increased microbial diversity has been associated with dysbiosis in other cancer types and could reflect a disrupted microenvironment favoring tumor initiation or progression. Importantly, β-diversity analyses confirmed that microbial composition differs across anatomical sites, reinforcing the hypothesis that the prostate harbors a specific microbial ecosystem rather than reflecting contamination from adjacent niches [[13], [14], [15], [16], [17], [18]].

However, as with other studies involving low-biomass samples, low-level residual contamination cannot be completely excluded despite the use of negative extraction controls and standardized procedures throughout the analytical workflow. Nevertheless, the biological plausibility and previously reported association of some of the identified taxa with prostatic tissue and prostate disease, including *Cutibacterium acnes*, provide additional context supporting the interpretation of our findings.

In this context, several bacterial taxa were enriched in prostate biopsy samples of PCa patients, including *C. acnes*, *E. rectale*, *B*. *dorei*, and *R*. *torques*. Among these, *C. acnes* has been consistently reported in prostate tissue and is known to induce chronic inflammation and modulate immune responses, supporting its potential role in carcinogenesis [[20], [21], [22], [23], [24]]. The enrichment of *E. rectale*, a species previously implicated in inflammation-related colorectal carcinogenesis, suggests that similar mechanisms could be involved in prostate tumorigenesis, although this remains speculative [25]. Conversely, *L*. *reuteri*, enriched in non-PCa patients, has documented anti-inflammatory properties and may contribute to a protective microbial environment [26].

Our study has several strengths, including its prospective design, multicenter recruitment, comprehensive clinical annotation and the use of full-length 16S sequencing enabling species-level resolution. However, several limitations should be acknowledged. First, all patients received antibiotic prophylaxis prior to biopsy, which may have altered microbial composition. Indeed, although this exposure was uniform across groups its impact on microbial composition cannot be excluded. Secondly, as prostate biopsy is not ethically justified in healthy individuals without clinical indication, the non-PCa group consisted of men undergoing biopsy for suspected PCa who had no evidence of malignancy on histological examination. This clinically relevant comparator group may nevertheless include patients with benign prostatic abnormalities, including prostatitis or adenoma, which may influence prostate microbiota composition**.** Third, the cross-sectional design precludes assessment of temporal relationships between microbiota changes and cancer development. Finally, functional implications of the identified taxa remain unknown and require further investigations.

Overall, our findings support the existence of a distinct prostate microbiota and suggest that specific bacterial species may be enriched in prostate biopsies from PCa patients. These results should be interpreted cautiously and require validation in independent cohorts, as well as mechanistic studies to determine whether these microorganisms play a role in prostate cancer development or represent secondary changes in the tumor microenvironment. Future studies should validate these findings using targeted approaches such as species-specific qPCR and culturomics on prostate biopsy samples. Given the limited accessibility of prostate tissue, these approaches should also be extended to more readily accessible specimens, particularly urine and rectal samples, to investigate whether relevant microbial signatures can be detected non-invasively. Functional studies using *in vitro* and *in vivo* models will then be required to clarify whether the identified bacteria are involved in tumorigenesis or reflect secondary changes in the tumor microenvironment. Ultimately, these efforts may support the development of microbial biomarkers for prostate cancer.

## CRediT authorship contribution statement

**R. Lotte:** Writing – original draft, Validation, Supervision, Resources, Project administration, Investigation, Funding acquisition, Formal analysis, Data curation, Conceptualization. **A. Gaudart:** Visualization, Formal analysis, Data curation. **C. Cruzel:** Software, Methodology, Formal analysis. **S. Droupy:** Investigation, Data curation. **J.P. Lavigne:** Investigation, Data curation. **F. Bruyère:** Investigation, Data curation. **P. Lanotte:** Investigation, Data curation. **E. Lechevallier:** Investigation, Data curation. **G. Dubourg:** Investigation, Data curation. **P.E. Fournier:** Investigation, Data curation. **R. Haider:** Investigation, Data curation. **L. Lotte:** Investigation, Data curation. **K. Youssef:** Investigation, Data curation. **A. Emery:** Investigation, Data curation. **R. Madany:** Investigation, Data curation. **M. Payen:** Investigation, Data curation. **L. Pfauwadel:** Investigation, Data curation. **B. Tibi:** Investigation, Data curation. **Y. Ahallal:** Investigation, Data curation. **S. Zouari:** Investigation, Data curation. **I. Bentellis:** Investigation, Data curation. **E. Fontas:** Software, Methodology, Formal analysis, Conceptualization. **M. Durand:** Visualization, Investigation, Data curation, Conceptualization. **R. Ruimy:** Visualization, Validation, Investigation, Formal analysis, Conceptualization.

## Ethics approval and consent to participate

The trial (NCT03947515) was approved by the institutional ethics committee (IDRCB: 2019-A00741-56) and conducted in accordance with the Declaration of Helsinki. Written informed consent was obtained from all patients included in the study.

## Availability of data and materials

All data are available upon reasonable request.

## Funding

This work was supported by fundings from Ministère de la Santé et de la Prévention of France: Clinical Trial MICROPROSTK 2019 (NCT03947515; ID-RCB: 2019-A00741-56) to Romain Lotte.

## Declaration of competing interest

The authors declare that they have no known competing financial interests or personal relationships that could have appeared to influence the work reported in this paper.
