# Supplementary material for: Prostate tissue microbiota dysbiosis associated with prostate cancer: results from the MICROPROSTK multicenter study
Source: New Microbes New Infect. 2026 Oct 8;74:101862. doi: 10.1016/j.nmni.2026.101862 (PMC13682004; doi:10.1016/j.nmni.2026.101862)
Supplement: Supplementary file 1 [file mmc1.docx]

# **Supplemental Materials – Materials and Methods**

## Clinical Cohorts and Study Design

MICROPROSTK included men with or without biopsy-confirmed PCa. Ethics approval (IDRCB: 2019-A00741-56) and written informed consent were obtained. Recruitment occurred in six French hospitals (Nice University Hospital, AP-HM La Conception Hospital, Tours University Hospital, Nîmes University Hospital, Cannes General Hospital, Menton General Hospital) between February 2021 and June 2023.

## Patient Enrollment and Sample Collection

**Inclusion Criteria:** Suspicion of PCa based on DRE, PSA, or MRI.
**Exclusion Criteria:** Recent antibiotics intake (3 months), recent uro-genital surgery, contraindications to biopsy.

**Sample Collection:**

- **Urine (Day 1):** 10 mL collected post-DRE, centrifuged at 4,500 × g for 15 min, resuspended in 1 mL DNase/RNase-free water, stored at –80°C.
- **Rectal swabs (Day 2):** Collected before biopsy, vortexed, 1 mL transferred to sterile cryotubes, stored at –80°C.
- **Prostate biopsies (Day 2):** Two biopsies per patient: MRI-index lesion if present or random from each lobe. Homogenized at 24,000 rpm for 2 min; 1 mL suspension stored at –80°C.

All patients received a single oral dose of ciprofloxacin (500 mg) 1–2 h prior to biopsy.

## DNA Extraction

Performed at Genoscreen (Lille, France) using optimized protocols:

- **Rectal samples:** QIAamp Fast DNA Stool Mini Kit (Qiagen)
- **Prostate and urine samples:** NucleoSpin Tissue Mini Kit (Macherey Nagel)

DNA quantified with SybrGreen assay (Life Technologies).

For each sample type (prostate, urine, and rectal samples), DNA extraction was performed in five batches, with one negative extraction control included in each batch. These negative controls subsequently underwent the same full-length 16S rDNA PCR amplification and sequencing procedures as the clinical samples

## SMRT 16S rDNA Sequencing

**Amplification:** Full-length 16S rDNA (~1 400 bp) with primers F27 and R1492.
**Library Preparation:** End-repair, A-tailing, adapter ligation, primer annealing, polymerase binding, quantification with Qubit, pooled by concentration.
**Sequencing:** PacBio Sequel II/IIe HiFi SMRT platform (Novogene, UK).
**Processing:** ≥3 passes, ≥90% predicted accuracy, trimmed to 1 340–1 640 bp, barcodes/primers removed, ASVs generated.

## Bioinformatics and Statistical Analysis for microbiota analysis

- **Denoising:** DADA2 or deblur in QIIME2; sequences with <5 reads removed.
- **Taxonomic Assignment:** classify-sklearn algorithm (Naive Bayes classifier).
- **Normalization:** Rarefaction to minimum library size, total sum scaling applied.

**Diversity Metrics:**

- **α-diversity:** Shannon index.
- **β-diversity:** Weighted UniFrac distances, visualized by PCoA in R.

**Statistical Analysis:**

Microbial diversity and composition were compared between PCa and non-PCa groups for each sample type. Alpha diversity (Shannon index) was compared using the Kruskal–Wallis test. Beta diversity was assessed using weighted UniFrac distances and compared using permutational multivariate analysis of variance (PERMANOVA, Adonis function). Differences in relative abundance were evaluated using unpaired Student’s t-tests. Given the large number of comparisons performed, results from differential abundance analyses should be interpreted with caution.

**Supplemental Materials – Results**

## Urinary microbiota analysis

In total, 12 different phyla, 17 classes, 55 orders, 104 families, 228 genera, and 483 species were identified across all sequenced urinary microbiota samples. The taxonomic biodiversity of the urinary microbiota is lower than that of the prostatic microbiota, which corroborates the findings of a recently published study by Goncalves *et al.* (*Cancers*, February 2023).
The five most represented phyla in the patients’ urine, in decreasing order of relative abundance, are: *Firmicutes*, *Actinobacteriota*, *Proteobacteria*, *Bacteroidota*, and *Campylobacterota.* The ten most abundant genera, in decreasing order, are: *Streptococcus*, Staphylococcus, *Peptinophilus,* *Anaerococcus*, *Enterococcus*, *Prevotella*, *Ezakiella*, *Corynebacterium*, *Escherichia/Shigella*, and *Lactobacillus*. This overall taxonomic composition of the urinary microbiota of the 141 patients in our study is consistent with previous reports in the literature. Furthermore, the taxonomic composition of the urinary microbiota differs from that of the prostatic microbiota in our patients.
Regarding the differences in relative species abundance in urine between the two patient groups, the most important finding is that *Cutibacterium acnes*, contrary to what is observed in the prostatic microbiota, is found at significantly higher levels in the control group than in the prostate cancer group.

## Rectal microbiota analysis

In total, 14 different phyla, 25 classes, 75 orders, 136 families, 378 genera, and 892 species were identified across all sequenced fecal microbiota samples. The phyla *Firmicutes* and *Bacteroidota*, followed by *Proteobacteria*, were the most represented in our study, as is commonly observed in the general population (Eckburg *et al.,* Science, 2005). However, a lower diversity of the fecal microbiota was observed compared to data reported in the literature and to the prostatic microbiota in our study. This may be explained by the fact that fecal samples were collected by rectal swabbing after enema on the day of prostate biopsies (and were therefore not stool samples).

Numerous studies have already investigated the role of the gut microbiota and potential fecal biomarkers in PCa. Our study shows that the genus *Bacteroides* is significantly enriched in the cancer group, which confirms the findings of a recent meta-analysis published in February 2024, demonstrating that the relative abundance of *Bacteroides* is statistically higher in patients with PCa compared to control patients (Huang *et al*., BMC cancer, 2024). This result is particularly interesting given that certain *Bacteroides* species are known to promote carcinogenesis.
